# Elevated Serum Gamma‐Glutamyl Transferase as a Risk Factor for Frailty in Older Men: A Nationwide Population‐Based Study

**DOI:** 10.1002/jcsm.70034

**Published:** 2025-07-29

**Authors:** Min‐gu Kang, Hee‐Won Jung, Beom‐Jun Kim

**Affiliations:** ^1^ Department of Internal Medicine Chonnam National University Bitgoeul Hospital Gwangju South Korea; ^2^ Department of Medical Science Chonnam National University Medical School Gwangju South Korea; ^3^ Division of Geriatrics, Department of Internal Medicine Asan Medical Center, University of Ulsan College of Medicine Seoul South Korea; ^4^ Division of Endocrinology and Metabolism, Department of Internal Medicine Asan Medical Center, University of Ulsan College of Medicine Seoul South Korea

**Keywords:** biomarker, frailty index, gamma‐glutamyl transferase, oxidative stress

## Abstract

**Background:**

Oxidative stress is a key driver of accelerated ageing, and gamma‐glutamyl transferase (GGT), an essential enzyme involved in the metabolism of glutathione, a major antioxidant, plays a pivotal role in the generation of free radical species. This study aimed to explore the potential utility of circulating GGT as a biomarker of frailty, which reflects biological ageing and overall health status.

**Methods:**

This cross‐sectional, population‐based study included 2526 community‐dwelling adults aged 65 years and older, using data from the Korea National Health and Nutrition Examination Survey. Frailty was assessed using a deficit accumulation frailty index (FI) derived from 36 items encompassing physical, cognitive, psychological, and social domains. Participants were categorised as nonfrail (FI ≤ 0.15), prefrail (0.15 < FI ≤ 0.25), or frail (FI > 0.25). Serum GGT levels were determined using an enzymatic activity assay.

**Results:**

After adjusting for potential confounders including age, body mass index, socioeconomic status, lifestyle factors, and medical history, serum GGT levels were 26.0% higher in frail men than in nonfrail men (*p* = 0.010). Amongst men, serum GGT concentrations were positively correlated with the FI (*p* = 0.001), and each standard deviation increase in serum GGT was associated with a 1.36‐fold higher odds of frailty (*p* = 0.001). Additionally, older men in the highest GGT quartile exhibited a significantly higher FI and a 2.08‐fold increased odds of frailty compared to those in the lowest quartile (*p* = 0.010 and *p* = 0.019, respectively). In women, however, no significant association was observed between serum GGT levels and frailty.

**Conclusion:**

Elevated serum GGT levels were significantly associated with frailty in older men, suggesting their potential as a biomarker of biological ageing. Nonetheless, the cross‐sectional design precludes causal inference, and longitudinal studies are warranted to explore whether elevated GGT contributes to the onset or progression of frailty over time.

## 1 Introduction

Frailty in older adults is characterised by an increased vulnerability to external stressors, stemming from the decline in physiological reserves associated with ageing [1]. The severity of frailty reflects an individual's overall health status and functional capacity, emphasising the clinical importance of frailty assessment in predicting adverse outcomes, including geriatric syndromes and mortality [2]. Two principal models are commonly used to assess frailty: the phenotype model, which focuses on evaluating physical attributes based on specific operational criteria [3], and the cumulative deficit model, which captures age‐related deficits across multiple domains, including social, psychological, and cognitive factors [4]. Recent longitudinal studies have shown that the frailty index, derived from the cumulative deficit model, correlates with molecular markers of ageing, supporting its validity as a surrogate indicator of biological age [5]. Although frailty was traditionally regarded as a self‐perpetuating condition [3], emerging evidence suggests that its progression can be prevented or reversed through appropriate interventions such as nutritional support, physical exercise, and multidimensional strategies [6, 7]. These insights highlight the urgent need to develop reliable biomarkers for frailty, which could substantially advance future research and serve as a foundation for more effective interventions aimed at mitigating frailty and promoting healthy longevity.

Gamma‐glutamyl transferase (GGT) has traditionally been recognised as a biomarker of hepatic dysfunction and excessive alcohol consumption [8]. However, accumulating evidence suggests that GGT also plays a role in the pathogenesis of various chronic diseases, including cardiovascular disease, cancer, pulmonary inflammation, and neurological disorders [9]. Population‐based studies have demonstrated that elevated serum GGT levels are associated with increased risks of all‐cause and cardiovascular mortality, independent of liver disease or alcohol intake [10, 11]. Mechanistically, GGT contributes to oxidative stress by facilitating the generation of free radical species through the extracellular hydrolysis of glutathione, the principal intracellular antioxidant [12]. Oxidative stress, in turn, plays a pivotal role in the development of numerous age‐related conditions, such as cardiovascular disease, chronic obstructive pulmonary disease, chronic kidney disease, neurodegenerative diseases, and cancer [13]. Given its association with oxidative stress and involvement in a broad spectrum of chronic conditions, GGT is increasingly regarded as a systemic health marker rather than solely a hepatic enzyme. Although the pathophysiological mechanisms underlying frailty are complex, oxidative stress has been identified as one of its key contributors [14]. Despite this biological plausibility, few studies have explored how serum GGT levels relate to frailty. Moreover, sex‐based differences in circulating GGT concentrations and frailty prevalence are well documented [8, 15], suggesting that the GGT–frailty link may differ by sex. Accordingly, we conducted gender‐stratified analyses from the outset. This study is aimed at examining the relationship between serum GGT and the frailty index in a nationally representative sample of community‐dwelling older adults, with particular attention to sex‐specific patterns.

## 2 Methods

### 2.1 Study Population

**FIGURE 1:**
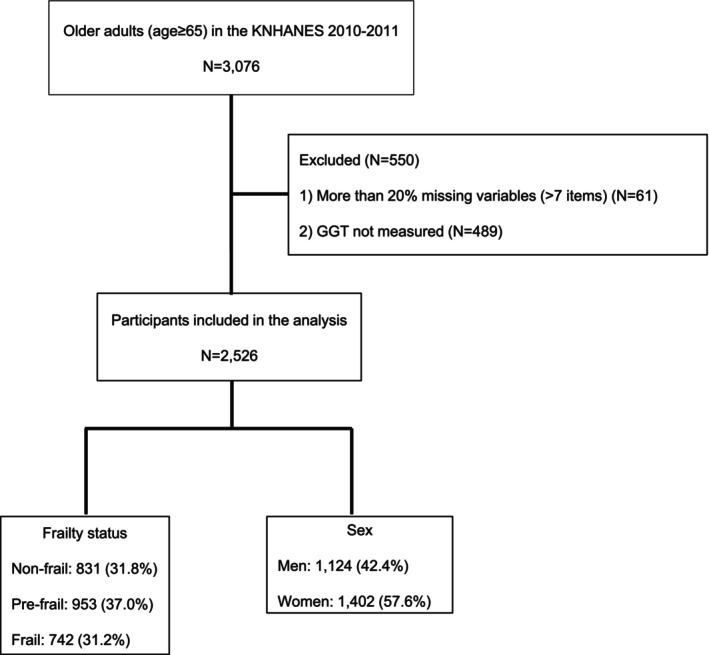
Flow diagram of the study participants. GGT, gamma‐glutamyl transferase.

This cross‐sectional study analysed data from the Korea National Health and Nutrition Examination Survey (KNHANES) conducted between 2010 and 2011. KNHANES is a nationally representative, ongoing surveillance programme launched in 1998 by the Korea Disease Control and Prevention Agency (KDCA) [16]. As of 2024, more than 20 waves of data collection had been conducted. It aims to comprehensively assess the health and nutritional status of the Korean population, track trends in health‐related behaviours and chronic disease prevalence, and provide evidence for national health policy planning. KNHANES employs a complex, multistage, stratified, probability‐cluster sampling design to represent the entire noninstitutionalised Korean population. Each year, approximately 50–60 primary sampling units (PSUs) are selected from census blocks or resident registration areas. From each PSU, 20–25 households are chosen through field surveys, and all individuals aged 1 year and older residing in these households are eligible to participate. Importantly, the availability of certain blood biomarkers varies by survey cycle. For example, serum GGT was only measured during the 2010–2011 cycle. Therefore, our study sample was restricted to this period. During this cycle, a total of 3076 individuals aged 65 years or older participated. For the present analysis, 2526 participants were included after excluding 61 individuals with more than 20% (i.e., over seven items) missing data on frailty assessment variables and 489 individuals without available GGT measurements (Figure 1). All participants provided written informed consent prior to participation. The dataset is publicly available in a fully de‐identified format. This study was approved by the Institutional Review Board of Chonnam National University Bitgoeul Hospital (IRB No. CNUBH‐2024‐019), which waived the requirement for additional informed consent, and was conducted in accordance with the Declaration of Helsinki.

### 2.2 Serum GGT Concentration Measurement

Blood samples for serum GGT measurement were collected from participants aged 10 years and older after obtaining informed consent. Following an overnight fast of at least 8 h, venous blood was drawn from the antecubital vein in the morning. The samples were immediately refrigerated and transported to the Central Testing Institute (Neodin Medical Inc., Seoul, Korea), where they were analysed within 24 h. Serum GGT concentrations were determined using an enzymatic activity assay with the Hitachi 7600 Automatic Analyser (Hitachi, Tokyo, Japan). The coefficient of variation for the assay was maintained at below 5%.

### 2.3 Frailty Index

The frailty index proposed by Rockwood et al. [4], which assesses the cumulative effects of psychosocial, medical, and functional deficits associated with ageing, was employed in this study. The specific frailty index used was developed using a standardised methodology [17] and was adapted from previously validated indices based on KNHANES data [15, 18, 19]. The index comprised 36 items surveyed between 2010 and 2011, generating a continuous score ranging from 0 (representing optimal health) to 1 (representing severe frailty). The included domains encompassed comorbidities (anaemia, arthritis, asthma, cancer, cardiovascular disease, diabetes, dyslipidemia, hypertension, and stroke), functional abilities (physical inactivity, low exercise capacity, limitations in activities of daily living, restrictions in social activities, inability to self‐care, and chewing difficulty), symptoms (pain or discomfort, fatigue, weight loss, depression, anxiety, suicidal ideation, and stress), and laboratory measurements (systolic and diastolic blood pressure, pulmonary function, haemoglobin, blood urea nitrogen, creatinine, total cholesterol, triglycerides, high‐density lipoprotein cholesterol, fasting glucose, vitamin D, and urine protein). Smoking status and body mass index (BMI) were also incorporated (Table S1). Based on established criteria, participants were classified into three categories: nonfrail (frailty index ≤ 0.15), prefrail (0.15 < frailty index ≤ 0.25), and frail (frailty index > 0.25) [20].

### 2.4 Frailty‐Related Factors Evaluation

Trained nurses measured blood pressure on the right arm using a mercury sphygmomanometer (Baumanometer Wall Unit 33(0850); W.A. Baum, Copiague, New York, United States) with an appropriately sized cuff after participants had been seated quietly for at least 5 min. Blood pressure was measured three times, and the mean of the second and third readings was used for analysis. Blood samples were collected during the survey period. BMI was calculated as weight in kilogrammes divided by height in metres squared (kilogramme per square metre). Socioeconomic status and lifestyle factors were assessed using self‐administered questionnaires. Household income was categorised into quartiles based on monthly earnings: < $680 (lowest), $680–$1360 (lower middle), $1360–$2230 (upper middle), and ≥ $2230 (highest). Educational attainment was classified into four levels: elementary school or lower, middle school, high school, and college or higher. Alcohol consumption was estimated based on self‐reported drinking frequency and the typical amount of alcohol consumed per drinking occasion. Smoking status was defined as having smoked five or more packs of cigarettes during one’s lifetime and currently smoking. Medical conditions were identified based on self‐reported physician diagnoses.

### 2.5 Statistical Analysis

To generate nationally representative estimates, complex sample analysis incorporating assigned sampling weights was employed. Data from the annual surveys were pooled, treating each year's sample as independent. Continuous variables were presented as means with standard errors (SEs), and categorical variables as frequencies with percentages. Baseline characteristics were compared using general linear models for continuous variables and cross‐tabulation methods for categorical variables. Clinically relevant and statistically significant covariates, including age, BMI, household income, education level, alcohol consumption, hypertension, diabetes, stroke, and cardiovascular diseases, were considered potential confounders. Differences in serum GGT levels according to frailty status, as well as differences in frailty index values across serum GGT quartiles, were assessed using general linear models. The association between serum GGT levels and the frailty index was evaluated through linear regression analysis, and the risks of prefrailty and frailty across serum GGT quartiles were examined using multivariable logistic regression analysis. All statistical tests were two‐tailed, with a significance threshold of *p* < 0.05. Analyses were performed using SPSS software, version 21.0 (IBM Corporation, Armonk, New York, United States).

## 3 Results

### 3.1 Baseline Characteristics of Study Participants

Table 1 summarises the baseline characteristics of 2526 participants aged 65 years and older. Amongst men, 470 (41.8%) were categorised as nonfrail, 429 (38.2%) as prefrail, and 225 (20.0%) as frail, with corresponding mean ages of 71.3, 71.4, and 73.2 years, respectively (*p* < 0.001). Amongst women, 361 (25.7%) were classified as nonfrail, 524 (37.4%) as prefrail, and 517 (36.9%) as frail, with mean ages of 71.9, 71.9, and 73.3 years, respectively (*p* < 0.001). In both sexes, advancing frailty status was associated with older age, lower household income, lower educational attainment, and a higher prevalence of hypertension, diabetes, stroke, and cardiovascular diseases (all *p* < 0.05). Additionally, alcohol consumption decreased significantly across frailty groups in men (*p* = 0.024), whereas no significant difference was observed in women (*p* = 0.480). Conversely, BMI increased significantly with increasing frailty severity in women (*p* < 0.001), whilst no significant trend was observed in men (*p* = 0.187).

**TABLE 1:** Baseline characteristics of the study participants according to frailty status.

Variables	Men (*N* = 1124)				Women (*N* = 1402)			
	Nonfrail (*N* = 470)	Prefrail (*N* = 429)	Frail (*N* = 225)	*p*	Nonfrail (*N* = 361)	Prefrail (*N* = 524)	Frail (*N* = 517)	*p*
Age (years), mean (SE)	**71.3 (0.2)**	**71.4 (0.3)**	**73.2 (0.4)**	**< 0.001**	**71.9 (0.3)**	**71.9 (0.2)**	**73.3 (0.3)**	**< 0.001**
Income quartile (%)				**0.001**				**< 0.001**
Low	**44.6%**	**45.0%**	**62.4%**		**48.0%**	**52.1%**	**65.6%**	
Midlow	**25.3%**	**32.5%**	**20.0%**		**24.9%**	**22.3%**	**18.0%**	
Midhigh	**18.1%**	**11.3%**	**8.8%**		**13.6%**	**13.5%**	**10.2%**	
High	**12.0%**	**11.2%**	**8.8%**		**13.5%**	**12.1%**	**6.3%**	
Education level (%)				**0.015**				**< 0.001**
1st	**45.5%**	**49.9%**	**55.8%**		**82.1%**	**83.1%**	**92.4%**	
2nd	**14.3%**	**18.7%**	**17.5%**		**7.4%**	**8.3%**	**5.2%**	
3rd	**21.7%**	**19.4%**	**20.8%**		**9.0%**	**7.6%**	**2.1%**	
4th	**18.6%**	**12.0%**	**6.0%**		**1.5%**	**1.0%**	**0.3%**	
Alcohol consumption (glasses/month), mean (SE)	**36.2 (3.6)**	**33.3 (3.3)**	**23.7 (3.2)**	**0.024**	3.4 (0.7)	2.4 (0.4)	2.5 (0.5)	0.480
Hypertension, *n* (%)	**216 (43.5%)**	**279 (64.2%)**	**161 (72.1%)**	**< 0.001**	**168 (51.6%)**	**360 (68.9%)**	**408 (80.9%)**	**< 0.001**
Diabetes, *n* (%)	**59 (11.3%)**	**109 (24.5%)**	**76 (34.6%)**	**< 0.001**	**38 (9.7%)**	**103 (20.3%)**	**159 (32.3%)**	**< 0.001**
Stroke, *n* (%)	**17 (3.8%)**	**23 (5.6%)**	**37 (18.1%)**	**< 0.001**	**6 (1.4%)**	**14 (2.8%)**	**45 (7.8%)**	**< 0.001**
Cardiovascular disease (MI, angina), *n* (%)	**23 (4.3%)**	**37 (8.6%)**	**32 (12.8%)**	**0.002**	**8 (1.6%)**	**22 (4.4%)**	**59 (10.6%)**	**< 0.001**
BMI (kg/m^2^), mean (SE)	22.9 (0.2)	23.3 (0.2)	23.2 (0.3)	0.187	**23.4 (0.2)**	**24.3 (0.2)**	**24.8 (0.2)**	**< 0.001**

*Note:* Continuous and categorial variables were compared using general linear model and crosstabs analyses in a complex sample analysis method, respectively. Bold numbers indicate statistically significant values.

Abbreviations: BMI, body mass index; SE, standard error.

### 3.2 Serum GGT Concentrations and Frailty Status

**FIGURE 2:**
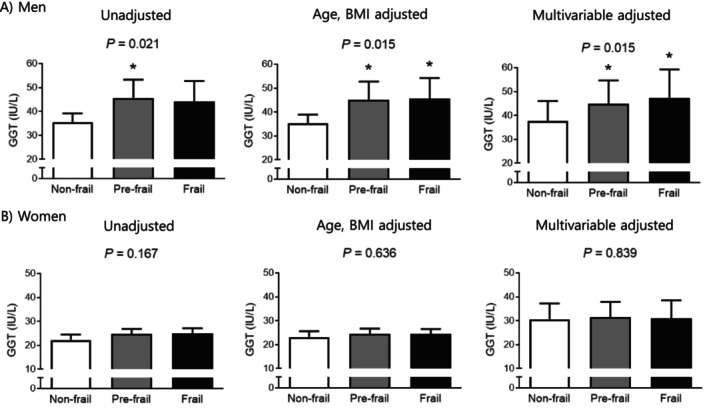
Differences in serum gamma‐glutamyl transferase levels according to the frailty status in (A) men and (B) women. The estimated means with 95% confidence intervals were generated and compared using general linear model analysis in a complex sample analysis method. Multivariable adjusted model: adjusted for age, body mass index, income, education level, alcohol consumption, hypertension, diabetes, stroke, and cardiovascular diseases. Asterisk indicates statistically significant difference from the nonfrail group. GGT, gamma‐glutamyl transferase.

Differences in serum GGT concentrations according to frailty status were analysed using a general linear model within a complex sample analysis framework (Figure 2). Amongst men, frail older adults exhibited significantly higher serum GGT levels compared to nonfrail individuals after adjustment for age and BMI (*p* = 0.031), and this association remained significant after further adjustment for socioeconomic and health‐related factors, including household income, education level, alcohol consumption, hypertension, diabetes, stroke, and cardiovascular diseases (*p* = 0.010). In women, however, no significant differences in serum GGT concentrations were observed across frailty status groups, irrespective of adjustments for potential confounders.

### 3.3 Association Between Serum GGT Levels and Frailty Index

Linear regression analyses were conducted to examine the independent association between serum GGT levels and the frailty index (Table 2). In men, higher serum GGT levels were consistently associated with a higher frailty index across all models, including the unadjusted model, the model adjusted for age and BMI, and the multivariable adjusted model controlling for household income, educational attainment, alcohol consumption, hypertension, diabetes, stroke, and cardiovascular diseases (*p* = 0.001 to 0.009). In women, no statistically significant association between serum GGT levels and the frailty index was observed across any of the adjustment models.

**TABLE 2:** Multiple linear regression analysis to determine whether serum gamma‐glutamyl transferase level is independently associated with frailty index.

Adjustment	Dependent variable: Frailty index		
	β	SE	*p*
Men			
Unadjusted	**0.000177**	**0.000067**	**0.009**
Age and BMI	**0.000198**	**0.000062**	**0.001**
Multivariable	**0.000191**	**0.000055**	**0.001**
Women			
Unadjusted	0.000346	0.000182	0.058
Age and BMI	0.000207	0.000180	0.251
Multivariable	0.000037	0.000162	0.822

*Note:* General linear model analysis was performed with frailty index as a dependent variable, and with serum gamma‐glutamyl transferase (IU/L) as an independent variable. Multivariable adjustment model includes age, body mass index, income, education level, alcohol consumption, hypertension, diabetes, stroke, and cardiovascular diseases as confounding factors. Bold numbers indicate statistically significant values.

Abbreviations: β, regression coefficient; BMI, body mass index; SE, standard error.

### 3.4 Risk of Frailty in Relation to Serum GGT Levels

Multiple logistic regression analyses were performed to assess the risk of prefrailty and frailty in relation to serum GGT levels (Table 3). In men, each standard deviation (SD) increase in serum GGT was significantly associated with higher odds of both prefrailty and frailty. For prefrailty, the associations remained significant across all models, including the unadjusted model (*p* = 0.010), the model adjusted for age and BMI (*p* = 0.012), and the multivariable adjusted model (*p* = 0.009). Similarly, for frailty, the crude odds ratio was 1.25 (*p* = 0.025), with the risk increasing by 30% and 36% in the age‐ and BMI‐adjusted and multivariable adjusted models, respectively (*p* = 0.009 and *p* = 0.001). In women, no statistically significant associations between serum GGT levels and the odds of prefrailty or frailty were observed across any of the adjustment models.

**TABLE 3:** Logistic regression analyses to determine the odds ratios for prefrail and frail status according to serum gamma‐glutamyl transferase level.

Adjustment	Prefrail		Frail	
	^a^Odds ratio (95% CIs)	*p*	^a^Odds ratio (95% CIs)	*p*
Men				
Unadjusted	**1.277 (1.060–1.538)**	**0.010**	**1.248 (1.028–1.515)**	**0.025**
Age and BMI	**1.280 (1.057–1.551)**	**0.012**	**1.298 (1.068–1.576)**	**0.009**
Multivariable	**1.283 (1.064–1.547)**	**0.009**	**1.364 (1.132–1.642)**	**0.001**
Women				
Unadjusted	1.164 (0.938–1.444)	0.168	1.176 (0.954–1.451)	0.129
Age and BMI	1.091 (0.891–1.337)	0.398	1.089 (0.885–1.339)	0.419
Multivariable	1.057 (0.856–1.307)	0.605	1.026 (0.821–1.283)	0.821

*Note:* Multivariable adjustment model includes age, body mass index, income, education level, alcohol consumption, hypertension, diabetes, stroke, and cardiovascular diseases as confounding factors. Bold numbers indicate statistically significant values.

Abbreviations: BMI, body mass index; CI, confidence interval.

^a^ Per standard deviation increment in serum gamma‐glutamyl transferase level (50.3 IU/L for men and 18.8 IU/L for women).

### 3.5 Threshold Effect of Serum GGT on Frailty Risk

To evaluate the possibility of a threshold effect, we conducted a series of piecewise linear regression analyses across a range of serum GGT cutoff values (15–40 IU/L; Table S2). Amongst men, these analyses revealed a consistent and statistically significant association between serum GGT and the frailty index in the higher GGT segments across various cutoff values. Notably, the association in the lower GGT segment became statistically significant starting at a cutoff of 30 IU/L, suggesting a potential threshold effect in the range of 25 to 30 IU/L. In contrast, no significant associations were observed in either the lower or higher segments amongst women, indicating a lack of threshold effect in this group.

**FIGURE 3:**
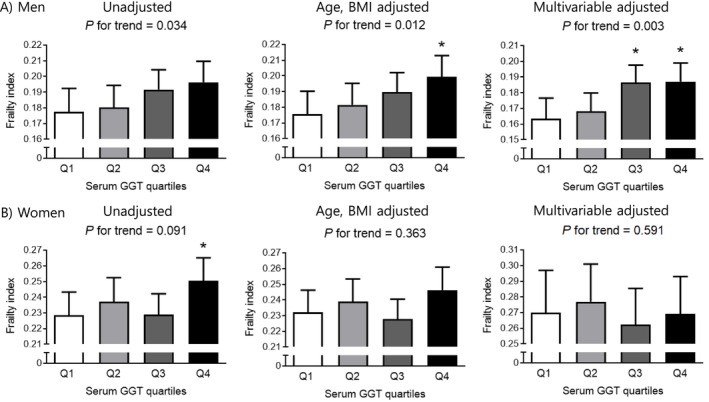
Differences in frailty index according to serum gamma‐glutamyl transferase quartiles in (A) men and (B) women. The estimated means with 95% confidence intervals were generated and compared using general linear model analysis in a complex sample analysis method. Multivariable adjusted model: adjusted for age, body mass index, income, education level, alcohol consumption, hypertension, diabetes, stroke, and cardiovascular diseases. Asterisk indicates statistically significant difference from the Q1 (lowest quartile). Q, quartile; GGT, gamma‐glutamyl transferase. GGT quartiles in men: Q1 = serum GGT ≤ 19 (IU/L), Q2 = 19 < serum GGT ≤ 26, Q3 = 26 < serum GGT ≤ 42, Q4 = serum GGT > 42. GGT quartiles in women: Q1 = serum GGT ≤ 14 (IU/L), Q2 = 14 < serum GGT ≤ 19, Q3 = 19 < serum GGT ≤ 26, Q4 = serum GGT > 26.

To enhance the clinical relevance of our findings, male and female participants were further categorised into sex‐specific quartiles based on their serum GGT concentrations (Figure 3). Amongst men, those in the third (Q3, 26 < serum GGT ≤ 42 IU/L) and highest quartile (Q4, serum GGT > 42 IU/L) exhibited a significantly higher frailty index compared to those in the lowest quartile (Q1, serum GGT ≤ 19 IU/L) in multivariable adjusted models (*p* = 0.013 and *p* = 0.010, respectively). In women, although the frailty index was initially higher in Q4 (serum GGT > 26 IU/L) compared to Q1 (serum GGT ≤ 14 IU/L) in the unadjusted model (*p* = 0.040), this association was no longer statistically significant after adjustment for age and BMI.

**FIGURE 4:**
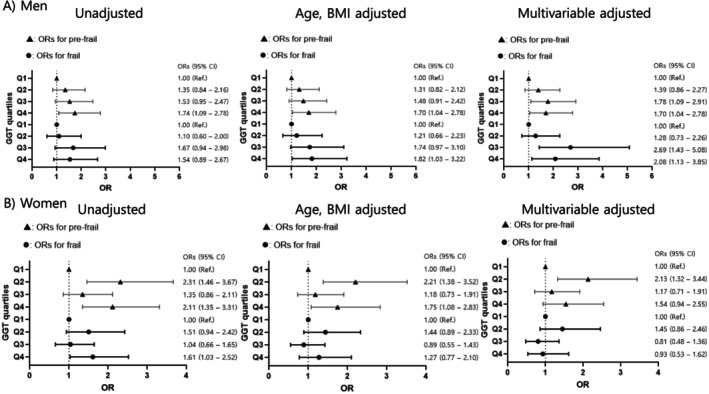
Logistic regression analyses to determine the odds ratios for prefrail and frail status according to serum gamma‐glutamyl transferase quartiles in (A) men and (B) women. Multivariable adjusted model: adjusted for age, body mass index, income, education level, alcohol consumption, hypertension, diabetes, stroke, and cardiovascular diseases. OR, odds ratio; CI, confidence interval; Q, quartile; GGT, gamma‐glutamyl transferase. GGT quartiles in men: Q1 = serum GGT ≤ 19 (IU/L), Q2 = 19 < serum GGT ≤ 26, Q3 = 26 < GGT ≤ 42, Q4 = serum GGT > 42. GGT quartiles in women: Q1 = serum GGT ≤ 14 (IU/L), Q2 = 14 < serum GGT ≤ 19, Q3 = 19 < serum GGT ≤ 26, Q4 = serum GGT > 26.

Logistic regression analyses were conducted to assess the odds of prefrailty and frailty across serum GGT quartiles (Figure 4). In men, participants in Q3 and Q4 had significantly higher odds of prefrailty compared to Q1 after adjustment for potential confounders (odds ratio = 1.78, *p* = 0.021, and odds ratio = 1.70, *p* = 0.035, respectively). Similarly, the odds of frailty were significantly higher in Q3 and Q4, with a 2.69‐fold and 2.08‐fold increase, respectively, in the multivariable adjusted model (*p* = 0.002 and *p* = 0.019, respectively). In women, the unadjusted model showed a 1.61‐fold increase in the odds of frailty in Q4 compared to Q1 (*p* = 0.037); however, this association was not statistically significant after adjustment for potential confounders.

## 4 Discussion

GGT is an enzyme primarily expressed in the liver, playing a critical role in glutathione metabolism and the regulation of oxidative stress. Traditionally, elevated serum GGT concentrations have served as clinical markers of hepatic dysfunction and excessive alcohol consumption. More recently, accumulating evidence has suggested that GGT is also implicated in a variety of metabolic and age‐related conditions [8, 9, 21]. In this large‐scale, population‐based study of community‐dwelling older adults aged 65 years and older, we observed that higher serum GGT levels were significantly associated with an increased frailty index, particularly amongst men. Serum GGT concentrations were notably higher in frail men compared to their nonfrail counterparts, and men in the highest GGT quartile showed an increased likelihood of both prefrailty and frailty, independent of potential confounding factors. These findings suggest a possible link between elevated circulating GGT levels and frailty risk in older men and support the potential utility of GGT as a candidate blood‐based biomarker for identifying individuals at higher risk.

Population ageing is a global phenomenon, and frailty is increasingly recognised as a critical clinical issue in geriatric care [15]. Two principal models are commonly used to define frailty: the phenotypic model and the cumulative deficit model [1]. Whilst both models are effective in predicting disease trajectories and responses to interventions, the Rockwood frailty index, derived from the cumulative deficit model, offers distinct advantages. By incorporating a wide range of health deficits, including medical conditions, functional impairments, and psychosocial factors, the frailty index provides a continuous and multidimensional measure of overall health status [4]. This gradation enhances its sensitivity to subtle changes in health, thereby improving its prognostic utility for adverse outcomes such as hospitalisation and mortality [22]. The KNHANES offers a nationally representative and comprehensive dataset that captures detailed health parameters, lifestyle factors, and clinical measures, making it an optimal resource for constructing the Rockwood frailty index and conducting large‐scale frailty research [16]. Although previous studies have demonstrated associations between circulating GGT levels and various chronic conditions that may predispose individuals to frailty, direct investigations examining the relationship between GGT and frailty remain limited. To address this gap, the present study leveraged nationally representative data to generate a comprehensive frailty index and provided novel evidence supporting the potential role of GGT as a biomarker for frailty risk.

High serum GGT levels may contribute to the development of frailty through multiple interconnected pathophysiological mechanisms. GGT promotes oxidative stress by facilitating the generation of free radicals via the extracellular hydrolysis of glutathione, the major intracellular antioxidant [8]. The resulting oxidative stress accelerates cellular ageing and impairs muscle function, contributing to sarcopenia, a central component of frailty [23]. Additionally, GGT plays a role in the progression of atherosclerosis by promoting the oxidation of low‐density lipoprotein cholesterol (LDL‐C) within atherosclerotic plaques [24]. Epidemiological studies have demonstrated positive associations between elevated GGT levels and the risk of coronary artery disease and cardiovascular disease [25, 26]. Elevated GGT levels have also been associated with heart failure, a condition that shares multiple pathophysiological pathways with frailty [27, 28]. Furthermore, GGT is positively correlated with inflammatory markers such as fibrinogen and C‐reactive protein (CRP), implicating its role in systemic inflammation [29]. Inflammation disrupts the balance between muscle protein synthesis and degradation, increases the expression of genes related to muscle atrophy, and interferes with hormones critical for muscle growth, ultimately impairing muscle metabolism and leading to sarcopenia [30, 31]. Inflammation is also closely associated with other age‐related conditions such as cardiovascular disease and diabetes mellitus, both of which contribute to the development of frailty [32]. Lastly, GGT remains a widely used marker of liver dysfunction, which itself is a recognised risk factor for frailty [33]. Indeed, when AST and ALT were added to the multivariable adjustment model, the association between serum GGT levels and frailty was attenuated (Table S3), although it remained statistically significant. This suggests that hepatic dysfunction may partially mediate the relationship between elevated GGT and increased frailty risk. Collectively, these diverse pathophysiological pathways suggest that GGT may serve as an important biomarker implicated in the progression of frailty syndrome.

In our study, a significant association between serum GGT levels and frailty was observed in men but not in women. Several plausible explanations may account for this sex‐specific discrepancy. First, although oxidative stress is considered a key mechanism through which GGT may contribute to frailty, women are generally less susceptible to oxidative damage due to lower production of reactive oxygen species and more robust endogenous antioxidant defences [34]. Oestrogen, in particular, has been shown to exert antioxidant properties, which may enhance oxidative resilience and mitigate frailty‐related biological pathways in women [35]. Second, serum GGT levels are typically lower in women than in men, which may lead to a narrower distribution across quartiles. These lower GGT levels are thought to reflect sex‐specific physiological regulation, potentially influenced by hormonal modulation [36]. Additionally, lower rates of alcohol consumption amongst women may further contribute to reduced hepatic GGT activity. Finally, metabolic and hormonal differences may also modulate the relationship between GGT and frailty. For example, women more commonly exhibit peripheral (gluteofemoral) fat accumulation, which is less metabolically active and less proinflammatory than the central (visceral) adiposity more prevalent in men [37]—a pattern that may influence systemic metabolic stress and its contribution to frailty. Collectively, these factors may attenuate the impact of elevated GGT on frailty risk amongst women.

To assess whether the null findings in women could be attributed to insufficient statistical power, we conducted post hoc power analyses. The results indicated that the study was sufficiently powered to detect modest associations, suggesting that the lack of statistical significance likely reflects a true absence of association rather than a limitation in sample size or analytical sensitivity.

To enhance the clinical interpretability of our findings, we adopted two complementary analytical approaches. First, we evaluated the association between serum GGT and frailty using standardised effect sizes. Specifically, in our logistic regression models, we estimated the odds ratios for frailty per one SD increase in serum GGT. This approach allows for meaningful comparisons across biomarkers with differing units and biological distributions—for example, GGT (international units per litre), high‐sensitivity CRP (milligrammes per litre), and uric acid (milligrammes per decilitre). In our study, amongst older men, each 1 SD increase in serum GGT was associated with a 36% higher odds of frailty (adjusted odds ratio = 1.36). This effect size is notably stronger than previously reported estimates for other frailty‐related biomarkers, such as hsCRP (odds ratio = 1.18 per 1 SD) [19] and uric acid (odds ratio = 1.22 per 1 SD) [18], suggesting that serum GGT may better capture systemic physiological stress relevant to frailty. Second, to further improve clinical relevance, we assessed frailty risk across GGT quartiles. Amongst older men, individuals in the third quartile and above (i.e., > 26 IU/L) exhibited significantly higher frailty index scores and increased odds of frailty compared to those in the lowest quartile. This threshold may provide clinicians with a practical reference for identifying individuals at higher risk of frailty who may benefit from closer monitoring or preventive strategies.

In the context of oxidative stress‐related frailty biomarkers, serum GGT offers several notable advantages. Biologically, GGT plays a central role in glutathione metabolism and the regulation of oxidative balance, and its pro‐oxidant properties have been linked to multiple age‐related conditions. Whilst other candidate biomarkers—such as interleukin‐6 (IL‐6), tumour necrosis factor‐α (TNF‐α), and derivatives of reactive oxygen metabolites—have been proposed in frailty research [38], their clinical use is often limited by high assay costs, specialised equipment requirements, and limited availability in routine settings. For example, IL‐6 and TNF‐α typically require ELISA‐based methods or high‐sensitivity multiplex assays, which may not be feasible for large‐scale implementation in clinical or community‐based screening. In contrast, GGT is widely available as part of standard liver function panels, can be measured through simple and cost‐effective enzymatic assays, and is already integrated into routine laboratory workflows worldwide. This makes it a highly practical candidate for translation into population‐level frailty screening, particularly in resource‐limited environments.

The primary strength of this study lies in the use of complex sample analysis methods incorporating assigned weights, which enabled the generation of nationally representative estimates and enhanced the generalisability of the findings. Additionally, the large sample size allowed for adjustment for a broad range of potential confounders, thereby increasing the statistical robustness of the results. Importantly, this is the first study to examine the association between serum GGT levels and frailty using a comprehensive deficit‐accumulation frailty index within a nationally representative cohort of older adults. Unlike mortality or disease‐specific endpoints, frailty captures multidimensional physiological decline and vulnerability, reflecting systemic ageing. By lever ageing the KNHANES dataset—a rigorously sampled, population‐based cohort—we were able to assess this relationship in a real‐world context with high external validity. Our findings extend the clinical significance of serum GGT beyond its traditional role as a hepatic enzyme, highlighting its potential as a biomarker for cumulative health deficits and biological ageing in older adults.

Despite these strengths, several limitations should be acknowledged. The most important limitation of this study is its cross‐sectional design, which precludes the ability to determine causality between serum GGT levels and frailty. It remains unclear whether elevated GGT contributes to the development of frailty or is a consequence of frailty‐related physiological changes. Second, the lack of oxidative stress and inflammatory biomarkers, such as CRP and IL‐6, in the KNHANES dataset limits our ability to explore the mechanistic pathways linking GGT to frailty. Future studies incorporating these biomarkers are needed to clarify whether the observed associations are mediated by systemic inflammation or oxidative stress. Third, several covariates, including alcohol intake, smoking status, and medical history, were based on self‐reported data, which may be prone to recall or reporting bias. Fourth, in the KNHANES dataset, serum GGT and body composition data were not collected during the same survey cycle, precluding a direct evaluation of the association between GGT levels and fat or muscle mass. Further studies integrating both biochemical markers and musculoskeletal or adiposity measures would be of particular interest. Fifth, because serum GGT was available only during the 2010–2011 cycle in KNHANES, the dataset may not fully reflect current trends in frailty prevalence or public health behaviours. Moreover, as the study population consisted exclusively of Korean older adults, given that serum GGT levels and frailty risk may be influenced by ethnicity, genetic background, dietary habits, and lifestyle factors, the generalisability of our findings to other populations should be confirmed through additional research. The exclusion of participants with missing GGT or frailty data could also be a limitation, potentially introducing selection bias. Lastly, although we adjusted for a wide range of covariates, there remains a possibility of residual confounding from uncontrolled variables that could influence serum GGT levels and/or frailty risk.

In conclusion, this nationally representative study of community‐dwelling older adults aged 65 years and older demonstrated that elevated circulating GGT levels are significantly associated with increased frailty in men, as assessed by a multidimensional frailty index encompassing physical, cognitive, psychological, and social domains. Whilst these findings suggest that GGT may serve as a potential biomarker for identifying older men at higher risk of frailty, the cross‐sectional design of the study limits causal inference. Therefore, longitudinal studies are needed to determine the temporal relationship between GGT levels and frailty development, as well as to assess whether interventions targeting GGT reduction could mitigate oxidative stress, slow biological ageing, and reduce frailty risk.

## Ethics Statement

The authors of this manuscript certify that they comply with the ethical guidelines for authorship and publishing in the *Journal of Cachexia, Sarcopenia and Muscle* [39].

## Conflicts of Interest

The authors declare no conflicts of interest.

## Supporting information


**Table S1.** Variables included in the frailty index
**Table S2.** Piecewise linear regression results for various serum gamma‐glutamyl transferase cutoff values in relation to frailty index.
**Table S3.** Logistic regression analyses to determine the odds ratios for prefrail and frail status according to serum gamma‐glutamyl transferase level
