# Supplementary material for: Elevated Serum Gamma‐Glutamyl Transferase as a Risk Factor for Frailty in Older Men: A Nationwide Population‐Based Study
Source: J Cachexia Sarcopenia Muscle. 2025 Jul 29;16(4):e70034. doi: 10.1002/jcsm.70034 (PMC12304734; doi:10.1002/jcsm.70034)
Supplement: Supplementary file 1 — Table S1. Variables included in the frailty index Table S2. Piecewise linear regression results for various serum gamma‐glutamyl transferase cutoff values in relation to frailty index. Table S3. Logistic regression analyses to determine the odds ratios for prefrail and frail status according to serum gamma‐glutamyl transferase level [file JCSM-16-e70034-s001.docx]

**Supplementary Table 1.** Variables included in the frailty index

|  | Items |
| --- | --- |
| Comorbidities | 1. Anemia 2. Arthritis 3. Asthma 4. Cancer 5. Cardiovascular disease (myocardial infarction, angina) 6. Diabetes 7. Dyslipidemia 8. Hypertension 9. Stroke |
| Functional abilities | 1. Activities of daily living limitation 2. Inactivity 3. Difficulty in exercise 4. Difficulty in self-care 5. Difficulty in social activity 6. Chewing difficulty |
| Signs and symptoms | 1. Anxiety 2. Depression 3. Fatigue 4. Pain or discomfort 5. Stress 6. Suicidal ideation 7. Weight loss (weight loss of 3 kg or more in the prior year) |
| Laboratory values | 1. Systolic blood pressure (>160 mmHg or <90 mmHg) 2. Diastolic blood pressure (>90 mmHg or <50 mmHg) 3. Hemoglobin (>18 g/dL or <11 g/dL) 4. Blood urea nitrogen (>20 mg/dL or <7 mg/dL) 5. Creatinine (>1.2 mg/dL or <0.6 mg/dL) 6. Fasting glucose (>250 mg/dL or <90 mg/dL) 7. Fasting cholesterol (total cholesterol > 270 mg/dL or < 135 mg/dL) 8. High-density lipoprotein cholesterol (<40 mg/dL) 9. Triglyceride (>200 mg/dL) 10. Vitamin D (>50 ng/ml or <12 ng/ml) 11. Proteinuria (urine dipstick test positive) 12. Pulmonary function test abnormality |
| Additional items | 1. Body mass index (<18.5 kg/m^2^ or >25 kg/m^2^) 2. Current smoking |

**Supplementary Table 2.** Piecewise linear regression results for various serum gamma-glutamyl transferase cutoff values in relation to frailty index.

| Cutoff (IU/L) | GGT_low | | | GGT_high | | |
| --- | --- | --- | --- | --- | --- | --- |
|  | β | SE | *P* value | β | SE | *P* value |
| Men | | | | | | |
| 15 | 0.000506 | 0.003220 | 0.875 | **0.000191** | **0.000055** | **0.001** |
| 20 | 0.001319 | 0.001327 | 0.321 | **0.000180** | **0.000057** | **0.002** |
| 25 | 0.001350 | 0.000762 | 0.077 | **0.000162** | **0.000060** | **0.007** |
| 30 | **0.001129** | **0.000547** | **0.040** | **0.000147** | **0.000063** | **0.020** |
| 40 | **0.000679** | **0.000343** | **0.049** | **0.000141** | **0.000068** | **0.038** |
| Women | | | | | | |
| 15 | 0.001643 | 0.002174 | 0.450 | 0.000001 | 0.000162 | 0.994 |
| 20 | -0.000364 | 0.001058 | 0.731 | 0.000068 | 0.000175 | 0.696 |
| 25 | -0.000043 | 0.000678 | 0.950 | 0.000050 | 0.000189 | 0.794 |
| 30 | 0.000036 | 0.000521 | 0.945 | 0.000037 | 0.000204 | 0.857 |
| 40 | 0.000132 | 0.000390 | 0.736 | -0.000010 | 0.000229 | 0.966 |

Bold numbers indicate statistically significant values. GGT; gamma-glutamyl transferase.

**Supplementary Table 3.** Logistic regression analyses to determine the odds ratios for pre-frail and frail status according to serum gamma-glutamyl transferase level

| Adjustment | Pre-frail | *P* value | Frail | *P* value |
| --- | --- | --- | --- | --- |
|  | ^*^Odds ratio (95% CIs) |  | ^*^Odds ratio (95% CIs) |  |
| Men |  |  |  |  |
| Unadjusted | **1.277 (1.060-1.538)** | **0.010** | **1.248 (1.028-1.515)** | **0.025** |
| Age and BMI | **1.280 (1.057-1.551)** | **0.012** | **1.298 (1.068-1.576)** | **0.009** |
| Multivariable | **1.283 (1.064-1.547)** | **0.009** | **1.364 (1.132-1.642)** | **0.001** |
| Additionally including AST and ALT | **1.295 (1.063-1.577)** | **0.011** | **1.293 (1.044-1.602)** | **0.019** |
| Women |  |  |  |  |
| Unadjusted | 1.164 (0.938-1.444) | 0.168 | 1.176 (0.954-1.451) | 0.129 |
| Age and BMI | 1.091 (0.891-1.337) | 0.398 | 1.089 (0.885-1.339) | 0.419 |
| Multivariable | 1.057 (0.856-1.307) | 0.605 | 1.026 (0.821-1.283) | 0.821 |
| Additionally including AST and ALT | 1.087 (0.854-1.384) | 0.498 | 1.062 (0831-1.356) | 0.630 |

Multivariable adjustment model includes age, body mass index, income, education level, alcohol consumption, hypertension, diabetes, stroke, and cardiovascular diseases as confounding factors. Bold numbers indicate statistically significant values. CI, confidence interval; BMI, body mass index; AST, aspartate aminotransferase: ALT, alanine aminotransferase. ^*^Per standard deviation increment in serum gamma-glutamyl transferase level (50.3 IU/L for men and 18.8 IU/L for women).
